# Development of a mobile application for health education about sepsis

**DOI:** 10.1590/1980-220X-REEUSP-2022-0269en

**Published:** 2023-06-05

**Authors:** Jhenyff de Barros Remigio Limeira, Vanessa de Carvalho Silva, Nelson Miguel Galindo, Cynthia Roberta Dias Torres Silva, Valdeilson Lima de Oliveira, Ana Carla Silva Alexandre

**Affiliations:** 1 Universidade Federal de Alagoas, Programa de Pós-Graduação em Enfermagem, Maceió, AL, Brazil.; 2 Instituto Federal de Pernambuco, Pesqueira, PE, Brazil.

**Keywords:** Mobile Applications, Sepsis, Patient Safety, Educational Technology, Health Promotion, Health Education, Aplicaciones Móviles, Sepsis, Seguridad del Paciente, Tecnología Educacional, Promoción de la Salud, Educación para la Salud, Aplicativos móveis, Sepse, Segurança do Paciente, Tecnologia Educacional, Promoção da Saúde, Educação em Saúde

## Abstract

**Objective:**

To develop and validate a mobile application for health education about sepsis.

**Method:**

Methodological study composed of two stages. Firstly, information from the Latin American Sepsis Institute and Global Sepsis Alliance was used, followed by putting together, design and layout of the application, according to the agile development model proposed by Sommerville. In the second stage, content validation occurred with 20 health professionals with expertise in the areas of intensive care and sepsis, from the use of the Instrument for Validation of Educational Content in Health through analyzing objectives, structure and relevance; and it was considered valid the item with a minimum agreement of 80%, by binomial test.

**Results:**

The app presents 15 screens that encompass prevention measures, recognition and early identification of sepsis, illustrated by interactive images. Out of the 18 items evaluated in the validation process, the minimum agreement obtained was 0.95 and the average validation index was 0.99.

**Conclusion:**

The application was developed and considered valid by the referees regarding contents. Thus, it is an important technological resource for health education in prevention and early identification of sepsis.

## RESUMO

**Objetivo:**

Desenvolver e validar aplicativo móvel para educação em saúde sobre sepse.

**Método:**

Estudo metodológico composto por duas etapas. Na primeira, foram utilizadas informações do Instituto Latino-Americano de Sepse e Global Sepsis Alliance, seguidos da montagem, design e layout do aplicativo, conforme modelo de desenvolvimento ágil proposto por Sommerville. Na segunda etapa, ocorreu a validação do conteúdo com 20 profissionais de saúde com expertise nas áreas de terapia intensiva e sepse, a partir da utilização do Instrumento de Validação de Conteúdo Educativo em Saúde que analisa objetivos, estrutura e relevância e foi considerado válido o item com concordância mínima de 80%, por teste binomial.

**Resultados:**

O aplicativo apresenta 15 telas que contemplam medidas de prevenção, reconhecimento e identificação precoce de sepse, ilustrados por imagens interativas. Dos 18 itens avaliados no processo de validação, a concordância mínima obtida foi 0,95 e o índice de validação médio foi 0,99.

**Conclusão:**

O aplicativo foi desenvolvido e considerado válido pelos juízes quanto ao conteúdo. Assim, consiste em importante recurso tecnológico para educação em saúde que contemple a prevenção e identificação precoce da sepse.

## RESUMEN

**Objetivo:**

Desarrollar y validar una aplicación móvil para la educación sanitaria sobre la sepsis.

**Método:**

Estudio metodológico compuesto por dos etapas. En la primera, se utilizó información del Instituto Latinoamericano de Sepsis y de la Global Sepsis Alliance, seguida del montaje, diseño y diagramación de la aplicación, según el modelo de desarrollo ágil propuesto por Sommerville. En la segunda etapa, se realizó la validación del contenido con 20 profesionales de la salud expertos en las áreas de cuidados intensivos y sepsis, a partir del uso del Instrumento de Validación de Contenidos Educativos en Salud que analiza objetivos, estructura y relevancia y se consideró válido el ítem con un acuerdo mínimo de 80%, mediante prueba binomial.

**Resultados:**

La app presenta 15 pantallas que contemplan medidas de prevención, reconocimiento e identificación precoz de la sepsis, ilustradas por imágenes interactivas. De los 18 ítems evaluados en el proceso de validación, el acuerdo mínimo obtenido fue de 0,95 y el índice medio de validación fue de 0,99.

**Conclusión:**

La aplicación ha sido desarrollada y considerada válida en lo que respecta al contenido por los jueces. Por lo tanto, es un importante recurso tecnológico para la educación sanitaria en la prevención e identificación precoz de la sepsis.

## INTRODUCTION

Sepsis is the set of organ dysfunctions secondary to ­unbalanced host responses to infection. Sepsis is among the critical and fatal diseases of greatest concern for public health worldwide, especially for the high mortality rate in Intensive Care Units^(1,2)^.

The context that contributes to greater aggravation of clinical cases of sepsis is related to late diagnosis, which suggests the need for early recognition and management. This reality can be verified by observing that 90.5% of deaths reported for sepsis as the underlying cause occurred in the hospital environment^(3,4,5)^.

Raising awareness and sensitization of the general ­population occurs through informative and educational strategies directed to the prevention and identification of sepsis. Therefore, scientific knowledge for health communication is essential in the process of improving the quality of care and changing the scenario, since it directly impacts the reduction of morbidity and mortality^(6)^.

The educational intervention effectively reduces the occurrence of sepsis, and collaborates in preventive actions from primary health care to timely management in specialized care^(7,8,9)^. In this context, we highlight the growth in the production of educational technologies in health that contribute to the improvement of care and enhance the construction of knowledge and empowerment of users. Presently, mobile apps represent a relevant instrument in the collaboration of innovative and informative educational learning^(9,10,11,12)^.

Information technology becomes a promising alternative for the expansion of access and improvement of health services. By fostering increased credibility, accuracy and organization, it reduces the proportion of errors, repetition of services and waiting time, which culminates in effective, efficient and cost-effective operation^(13)^.

There are already apps created for sepsis diagnosis, for example, Sepsis Clinical Guide®, Sepsis App 3.0® and Sepsis Code®, available in the Google Play app library, that allow calculation of scores, clinical and treatment information queries. However, a gap is observed regarding software aimed at education and general recognition of sepsis by laypersons.

Given the need to educate, inform and direct potential users with sepsis, from the identification of signs, symptoms and risk factors, with accessible language and guidance of safe content, this study aimed to develop and validate mobile application for health education on sepsis.

## METHOD

### Study Design

This is a methodological study conducted applied to the construction and content validation of the app for early identification of sepsis for general population, conducted between March 2019 and June 2021.

The study population consisted of healthcare professionals with expertise in the areas of intensive care and sepsis. The Sepsis Quick Guide app was developed and validated in a virtual environment.

The eligible health professionals met the following inclusion criteria: specialization in the intensive care field, assisting in research or teaching on the subject or with professional training on sepsis. The exclusion criterion was sending the data collection instrument with incomplete data.

### Stages of Development

The first step consisted in the development of the app, based on the software engineering model of agile development, by allowing a constant and simultaneous construction, evaluation and adaptation of elements according to the selection of content, design and layout^(14)^.

The selection of content and textual construction was done based on the recommendations of the Latin American Sepsis Institute (ILAS in the Portuguese acronym) and Global Sepsis Alliance (GSA), considering concepts and definition of sepsis, as well as the signs and importance of early recognition^(15,16)^. The application was developed on the free Android Studio platform with 15 screens and Java programming that included Application Programming Interface (APIs)^(19–28)^. For usability and access to secondary screens, we included buttons available on the platform itself as well as images created in the *Inkscape* program using vectors available on *Freepik*.

The screens were created from the LibreOffice Impress sketches, with adjustments to the content and characteristics that were improved during development, until the final version to be programmed. The predominant colors were shades of orange, blue, and black, with a relaxed font and varying sizes from 18 to 35.

Resulting data were analyzed using the software R®, version 3.1.1. The data analysis regarding the characterization of professionals was done descriptively, from absolute and relative frequency. The content validation data were analyzed item-by-item based on the proportion of agreement of the professionals. Moreover, the average proportion of the items was calculated from the sum of the proportions divided by the number of items, to obtain the general proportion of agreement of the professionals about the application. The binomial test was used to verify if the agreement was statistically equal or higher than 80%, which was the value used for the item to be considered valid^(17)^.

### Content Validation

At this stage, participants were selected by convenience, based on the recommendation of researchers from the Interdisciplinary Nursing Research Group who pointed out names and contact details of professionals with a profile eligible for the study, belonging to all regions of the country, which, through snowball sampling, indicated other professionals. Thus, an invitation was sent, via e-mail, to 72 professionals, and a response was obtained from 20, who made up the study sample.

After accepting to participate in the study, the evaluating referees were sent a link to download the application content, which could be viewed in two ways: through a demonstrative video or from images of the screens in PDF. In addition, it was sent in Survey format, an instrument developed in Google Forms, which had the Informed Consent Form (ICF), followed by the sections “Characterization of the evaluator” and “Validation of the content”.

The latter, in order to register the professionals’ evaluation, had the Instrument for Health Educational Content Validation (IVCES in the Portuguese acronym), which is a validated instrument based on three domains: 1) Objectives, which addresses purposes, goals, or purposes presented by the software; 2) Presentation, directed to the organization, strategy, coherence, and sufficiency of the application structure; and 3) Relevance, related to the impact, significance, motivation, and interest that the content presents. The presentation of the IVCES answer possibilities is arranged by means of a Likert scale, with the options 1 (disagree), 2 (partially agree), and 3 (totally agree). In addition, the instrument offers space to register considerations or suggestions.

### Ethical Aspects

The research was cleared by the ethics committee of the Autarquia Educacional de Belo Jardim- PE under CAAE: 05050918.5.0000.5189, following ethical aspects determined in Resolution 466/2012 of the National Health Council (CNS in the Portuguese acronym) and a patent application was filed with the National Institute of Industrial Property (INPI in the Portuguese acronym), according to registration number BR512020002407.

## RESULTS

The application was named as Guia Rapido da Sepse (Sepsis Quick Guide or GRS in the Portuguese acronym), is available for free download from Play Store and has 15 screens with layouts of two categories: the Frame, which has graphic elements inside it positioned at the top, bottom, right and left, and the Linear, which has its graphic elements positioned in horizontal and vertical sequences. After installing the application, the developed content can be viewed without an internet connection, with the exception of external content with information from reliable sites about sepsis.

**Figure 1:**
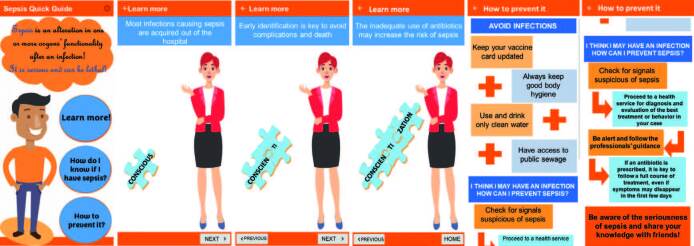
“Initial”, “Learn more”, and “How to prevent” screens of the Sepsis Quick Guide application. Pesqueira, PE, Brazil, 2021.

The home screen presents the concept of sepsis, providing options through buttons, for secondary parts that direct to the content frame: “Information”; “Learn more”; “How do I know if I have sepsis?”; and “How to prevent”. On the “Learn more” screen there is additional information to raise awareness about the occurrence and risks of sepsis and the importance of early identification. The “How to prevent” screen presents educational information about habits and attitudes that are fundamental for proper prevention (Figure 1).

**Figure 2:**
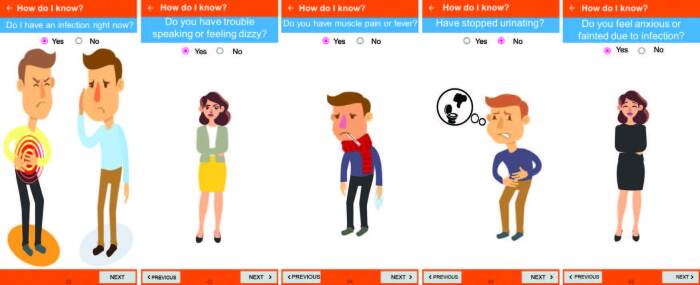
Screenshots “How to know if I have sepsis?” of the app Sepsis Quick Guide. Pesqueira, PE, Brazil, 2021.

The next screen “How to know if I have sepsis?” includes the identification of signs related to sepsis that may be suggestive for the user in order to seek health care. This screen presents eleven questions about signs and symptoms associated with risk factors, which feed an algorithm for the possibility of involvement by sepsis (Figure 2).

**Figure 3:**
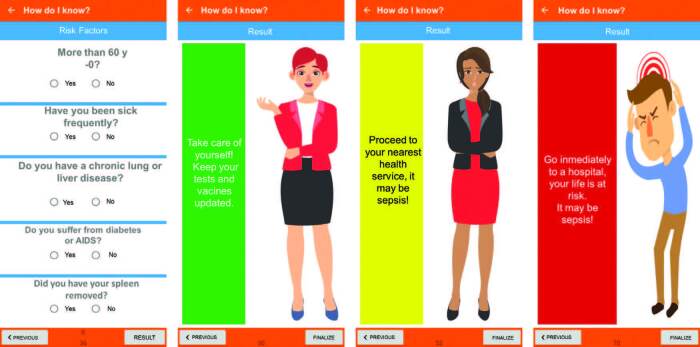
Screens with guidelines produced as the user fill in the screen “How do I know if I have sepsis?” of the Sepsis Quick Guide application. Pesqueira, PE, Brazil, 2021.

Using the answers filled in by the user, the algorithm associates the possibility of presence or absence of infection with signs and risk factors, scoring them according to their severity and possible association with sepsis, according to the recommendations of the ILAS protocol. At the end of the answers, according to the algorithm results, the application will present one of three possible orientations: ‘Please take care’; ‘It may be sepsis’; ‘Your life is at risk’ (Figure 3).

In addition, the “Information” screen has three buttons that lead the navigation to reference sites for sepsis, arranged in “Stop sepsis, save lives”, “American Sepsis Institute”, and “Global Alliance on Sepsis” buttons.

Professionals from 28 to 44 years old participated in the validation process, belonging to the Northeast (80%), Southeast (15%) and Midwest (5%). The most predominant education was nursing, with 15 (75%) professionals, followed by physiotherapy, with four (20%) and medicine with one (5%). Regarding the time of experience in the profession, nine (45%) professionals had three to 10 years of training in the area, and 11 (55%) from 10 to 20 years. Regarding clinical work in the ICU, 17 (85%) professionals had two to 10 years of experience in the sector and three (20%) from 13 to 17 years, especially in the adult ICU.

Regarding the experts’ degrees, a predominance of PhDs was identified, with eight (40%) professionals, followed by nine (45%) specialists and three (15%) masters. Regarding education directed to the ICU, nine (45%) had *Lato Sensu* specialization, eight (45%) improved through training, four (20%) had *Stricto Sensu* specialization and two (10%) did residency in the area. It is noteworthy that three evaluators had more than one education. Regarding professional experience, 19 (95%) of the evaluators had more than one year of clinical practice in ICU, 18 (90%) said they had experience with assistance to patients with sepsis, 14 (70%) had already taught courses that addressed sepsis, 14 (70%) had participated in training and courses on sepsis and 11 (55%) had participated in the process of content validation as referee/evaluator of educational technologies in health.

Regarding the validation of the application, out of the five items assessed in the dimension “Objectives”, two had unanimous agreement and three had agreement of 95% of professionals, while in the 10 items assessed in the dimension “Structure/Presentation” and the three items assessed in the dimension “Relevance” had 100% agreement of professionals, as detailed in Table 1. The average agreement of the professionals was 99% for the 18 items evaluated, so that the application was considered valid as to content.

**Table 1:** Percentage of agreement among the content validation experts regarding the Sepsis Quick Guide (GRS) application. Pesqueira, Pernambuco, Brazil, 2021.

Items	Disagree %	Partially agree %	Fully agree %	I-CVI*	p^†^
**Objectives**					
Address proposed topic	5	10	85	0.95	0.878
Adequate to the teaching-learning process	0	25	75	1.00	1
Clears up doubts about the topic	0	15	85	1.00	1
Provides reflection on the theme	5	5	90	0.95	0.878
Encourages behavioral change	5	25	70	0.95	0.878
**Structure/Presentation**					
Appropriate language for the target audience	0	15	85	1	1
Language corresponds to the educational material	0	15	85	1	1
Interactive language, allowing active involvement in the educational process	0	15	85	1	1
Correct information	0	0	100	1	1
Objective information	0	15	85	1	1
Clarifying information	0	15	85	1	1
Necessary information	0	0	100	1	1
Logical sequence of ideas	0	5	95	1	1
Current topic	0	0	100	1	1
Appropriate text length	0	20	80	1	1
**Relevance**					
Stimulates learning	0	0	100	1	1
Contributes to knowledge in the area	0	5	95	1	1
Stimulates interest in the topic	0	20	80	1	1

Source: Data from research, 2021.

Caption: *Item Content Validity Index; †Binomial test.

In addition to the results obtained by the evaluation of agreement between the items analyzed in the application, we added the main suggestions and considerations of the referees for the achievement of quality content and the decisions made by the adequacy of the objective proposed by the technology.

Regarding the objectives, to broaden the understanding of the target audience regarding the screen “How to know if I have sepsis”, the following changes proposed by the referees were made: the information “associated with infection” was removed from the sentence “Have anxiety or loss of consciousness associated with infection?”, because the target audience could not assess whether the loss of consciousness is related to infection. On the screen “How to know if I have sepsis” there was the replacement of the information “feeling confused” to “perceiving yourself with disorganized thoughts” from the sentence “Are you talking with difficulty or feeling confused?”.

On the “How to prevent” screen, to make the prevention guidelines clearer, the referees suggested explaining why, resulting in: changing the phrase “Avoid infections” to “Habits that avoid infections”. All the orientations given on this screen have as a motive the decrease in the occurrence of contaminations and the direction to the health service. On the screen “How to know if I have sepsis”, about the term “low immunity”, information was added in parentheses “Get sick often/have some syndrome”, to identify the risk for immunity.

Finally, it was also suggested to review repeated verbs, use of verb locution and verb tenses that are not in the imperative and detailing of clinical signs, since the technology will be directed to laypeople, for example: Fever (>37.8°C). To meet this suggestion, the use of language throughout the application was reviewed by a linguistic specialist and the signs were detailed in parentheses.

## DISCUSSION

Information and Communication Technologies (ICTs) allow to produce tools capable of informing and connecting people of different educational levels from digital electronic resources with information exchange functions, either in real time or in offline mode only by consultation^(18)^. ICTs may be used in a very wide and efficient way in education, even more assertively whenever is combined with health. These possibilities can provide the same or different information to individuals in different places, and when applied to projects for the general population, its reach may be wider than if compared to some health education activity at the local level using printed materials, for example.

Thus, ICTs applied to lay people with the purpose of raising awareness have been great motivators in health promotion actions and, as an example, there is an application developed for pregnant women to provide information and health actions to strengthen the autonomy of users during prenatal care, which influenced the decision making of most of them based on the knowledge acquired by technology^(19)^.

WHO recommends raising awareness of the population about the aggravations of infectious diseases and the aspects related to sepsis, in order to promote health education through the prevention of new cases^(20)^. In this context, the use of mobile application in health has proven to be an excellent tool to stimulate knowledge of easy access and simplified form, which infers and corroborates the relevance of development and validation of the application fruit of this study^(21)^.

Knowledge represents the first factor of awareness, by understanding the impact of sepsis and the need for effective infection prevention actions, and need to be extended to the population and health professionals^(1)^. Data coming from public and private hospitals in Brazil, published by ILAS reinforce the importance of access to information for the general population, justified with the analysis of the impact of sepsis, since in 2017, the year of recognition of sepsis as a public health problem by WHO, there were 11,941 cases of sepsis and septic shock, with lethality of both equal to 3,457 deaths. In 2019, the numbers were even higher, with over 15,000 cases and over 4,300 deaths^(20)^.

In addition to its large mortality rate, treating sepsis also leads to high expenses to the health system. In a study conducted in southern Brazil it is possible to observe that the total expenditure on the hospitalization of patients with sepsis was R$ 3,692,421.00 million, R$ 38 thousand per patient, and of these, 59% died^(22)^. It is expected that by applying the recommendations for prevention of infections with positive response, early identification and annual decrease in the number of cases and deaths from sepsis, in addition to the number of lives that will be saved, the costs of hospitalization associated with the disease and its complications can be reduced. These facts reinforce the need for multiplication of information about sepsis beyond health professionals.

A cross-sectional study conducted in Porto Alegre, Brazil, showed that among 1,986 persons going to public parks as well as companions of hospitalized patients, less than 20% of them knew about sepsis while more than 98% knew what is Acute Myocardial Infarction^(23)^. In this context, the content addressed in the application presented provides information necessary for knowledge, early identification and prevention of sepsis with screens on awareness and characterization of the problem, so that the application is an important resource for the multiplication of information on the subject.

As for the fact that the information presented is necessary, correct, provide stimulus to learning and deal with current issues, the application had unanimous agreement among professionals, thus highlighting the importance of professional experts in the rationale of this instrument. A similar result was obtained in the validation of an application focused on breastfeeding^(24)^. In this sense, it is highlighted the importance of the evaluation of health technologies carried out by a multiprofessional team that attributes to the application judgment from different professional perspectives, which have different attributions, knowledge and technical and scientific capabilities^(25)^.

The incorporation of information by health technologies for the civil society is encouraged, since it has intervention by habit implementation, from the public’s own health preference^(26)^. Compared with the technology presented, the GRS app presents graphic designs and use of language without technical terms related to sepsis and addresses content for awareness, knowledge and identification of sepsis and methods of prevention, this importance stands out because all health technologies should be idealized and operationalized focused on the public who will consume them.

The interactivity in the screens “How to know if I have sepsis?”, allows the leading role and participation of the user in the learning process, with elements associated with sepsis, with indication of severity associated with the set of infection, signs, symptoms, and risk factors. With the enforcement of access to information about sepsis for general awareness, the number of people with this knowledge can grow and encourage the adoption of preventive measures for diseases that cause infection in the community and hospital environment.

This can contribute to better adherence to infection prevention practices, which as presented in the “How to prevent” screen, can be actions such as proper hygiene, correct use of antibiotics, and access to basic sanitation. In addition, it is important that the suspicion and rapid recognition of sepsis are widespread, because the diagnosis can be made early and treatment started immediately to reduce the risk of death and expenses of the health system, these being highlighted as the main significant points to the use of the application and its future impacts.

The referees were selected from three regions of Brazil, with profiles considered necessary to the judgment, since sepsis, even presenting similar characteristics, socio-demographic issues can impact on its phases. The evaluators showed agreement to the GRS app as a resource to be used by lay people outside the hospital environment, through qualitative suggestions made in the evaluation. The accessible language that allows interaction may favor the adhesion of the population in the search for knowledge.

The consideration of semantic terms and to improve the description of prevention measures were suggested by the professionals and accepted by the researchers. In this sense, the use of health technology by society reveals the strengthening of practicality and technical-scientific expansion of care, especially when these go through strong judgment criteria. The resulting evaluations should be based on scientific evidence that makes them suitable for use in society^(27,28)^.

Another study of educational material validation also obtained a high proportion of agreement from professionals, with a result that infers viability of favorable use of the developed resources^(29)^. In this context, the evaluation process of educational technologies in health strengthens the technical-scientific expansion of care, since the educational technologies that are the target of the studies are more likely to be composed of correct content, understandable language, and to be based on scientific evidence, which increases the feasibility and adherence for use in society.

A limitation of the study is the evaluation in the early stages of the application, with validation directed only to the content. It is important to verify the appearance and usability of health technologies, especially regarding the use by the target audience of the technology.

This study contributes to the advances in nursing and health, since the application has feasibility to contribute to the access to information and allow the user to understand the severity of sepsis, as well as the importance of prevention and early identification as measures to reduce mortality, which makes the technology useful for various forms of health education inside and outside the hospital environment.

Nursing, as a professional group active in health education, can apply this application in the tripod teaching-research-extension so that this study can also encourage technological production in health care, in order to propose changes in the scenario of health needs from the replication of the method used.

## CONCLUSION

The educational material developed arises in the face of educational needs and prevention of sepsis of the general population, as well as the lack of technologies aimed at recognition of sepsis by lay people. The relevance of health education on the subject is ratified since ILAS and GSA provide content on their websites for the general population.

The GRS application was developed and provides information about the concept, severity, prevention, and early identification of sepsis. The content was validated and obtained a high proportion of agreement from experts in sepsis, regarding the objectives, structure and relevance. Therefore, the application is a technological resource, indicated for the general public, without training in health, and has compatible access to mobile devices being able to promote communication, education and awareness in health.
